# Prevalence of Potentially Modifiable Risk Factors in Persons At-Risk for Alzheimer’s Disease

**DOI:** 10.1093/geroni/igaf122.4264

**Published:** 2025-12-31

**Authors:** Zaldy Tan, Nabeel Qureshi, Drew Hirsch, Mitzi Gonzales, Sarah Kremen, Stephanie Bray, Anna Czarny, Nancy Sicotte

**Affiliations:** Cedars-Sinai Medical Center, Los Angeles, California, United States; Cedars-Sinai Medical Centers, Los Angeles, California, United States; Cedars-Sinai Medical Center, Los Angeles, California, United States; Cedars-Sinai Medical Center, Los Angeles, California, United States; Cedars-Sinai Medical Center, Los Angeles, California, United States; Cedars-Sinai Medical Center, Los Angeles, California, United States; Cedars-Sinai Medical Center, Los Angeles, California, United States; Cedars-Sinai Medical Centers, Los Angeles, California, United States

## Abstract

Addressing modifiable medical and lifestyle risk factors may reduce the incidence of Alzheimer’s disease and related dementias (ADRD). The Cedars-Sinai Memory & Health Aging Program (MHAP) promotes brain health via personalized risk profiling and risk reduction among asymptomatic adults aged 40 years and older at risk for ADRD. MHAP was launched with an email invitation to Cedars-Sinai Medical Center patients. Eligibility required age 40 or older, no cognitive or neurological diagnosis, and presence of two or more ADRD risk factors. Participants completed a 90-minute in-person visit assessing ADRD risk factors and cognitive performance. Of 123 enrolled patients, 84 (68.2%) had a family history of dementia or carried at least one ApoE4 allele. The mean age was 59.5 years; 63.1% were female. Most were White (77.4%) or Asian (9.5%), non-Hispanic (95.2%), and highly educated (91.7% college graduate or higher). Common risk factors included family history of ADRD in a first-degree relative (92.9%), weak social networks (52.4%), sleep disorder (51.2%), elevated LDL cholesterol (50%), and obstructive sleep apnea (50%). Among those completing the Montreal Cognitive Assessment (MoCA), the average score was 26.0, with frequent difficulties in delayed recall (91.9%), visuospatial/executive function (52.7%), language (36.5%), and attention (27.0%). Personalized risk profiling revealed high rates of modifiable ADRD risk factors, including sleep disorders, elevated cholesterol, and low physical and social activity. Targeted interventions addressing these factors may reduce future ADRD risk in at-risk populations.

